# A bioinformatic single-cell and structure-informed framework identifies a baicalin–CA2–keratinocyte state axis in atopic dermatitis

**DOI:** 10.1371/journal.pone.0356174

**Published:** 2026-09-11

**Authors:** Boyan Yang, Guilin Zhou, Jun Dai

**Affiliations:** 1 Department of Dermatology, The First People’s Hospital of Yibin. No. 16 Puhe East Road, Yibin, Sichuan, PR China; 2 Department of Dermatology, The Fourth People’s Hospital of Sichuan Province, No. 12, Chengshou East Street, Jinjiang District, Chengdu, Sichuan, PR China; Istituto Dermopatico dell’Immacolata (IDI)-IRCCS, ITALY

## Abstract

Atopic dermatitis (AD) is characterized by a self-reinforcing loop between epidermal barrier dysfunction and type 2-skewed inflammation; yet the most perturbed keratinocyte states and actionable epidermal targets remain incompletely defined. We integrated pharmacogenomic target mining, complementary machine-learning feature selection (LASSO and SVM-RFE), single-cell state–resolved perturbation analyses (Augur and scDist), and structure-based molecular modeling (molecular docking, MD simulation, and MM-PBSA free energy calculation) to prioritize candidate targets of baicalin in AD. CA2 emerged as a convergent epidermal candidate; scRNA-seq analyses localized CA2-associated transcriptional differences to keratinocytes, with the keratinocyte compartment exhibiting the disease-associated strongest separability and transcriptomic distance, accompanied by enrichment of metabolic reprogramming, epithelial junction and barrier remodeling, and proliferative quiescence gene programs. Structure-based evaluation supported a computationally plausible baicalin–CA2 interaction, with an estimated MM-PBSA binding free energy of −22.082 kcal/mol. Collectively, these findings nominate a computationally supported “baicalin–CA2–Kcs9” axis as a hypothesis-generating framework for epidermal stratification and experimental prioritization in AD.

## Introduction

Atopic dermatitis (AD) is a common, chronic, relapsing inflammatory skin disease characterized by intense pruritus, sleep disturbance, and impairment of quality of life [1,2]. Despite major therapeutic advances, many patients experience incomplete control, recurrence after treatment withdrawal, or heterogenous responses, reflecting the biological complexity of AD across individuals and disease stages [3]. A central feature of AD is the tight coupling between epidermal barrier dysfunction and type 2-skewed inflammation: barrier disruption facilitates allergen and microbial penetration, while inflammation further impairs epidermal differentiation and barrier integrity, thereby creating a self-reinforcing loop [4]. This clinical and biological landscape motivates a more precise understanding of which epidermal cell states are most perturbed and which molecular targets are most actionable in AD [5].

Keratinocytes, the predominant cell type of the epidermis, are increasingly recognized not merely as passive structural components but as active regulators of cutaneous immunity and tissue remodeling [6]. Beyond forming the physical barrier through differentiation programs and junctional architecture, keratinocytes produce cytokines and chemokines including alarmins and recruitment signals, engage in stress-response and metabolic programs, and participate in crosstalk with immune and stromal cells that shapes inflammatory circuits in AD [6–9]. This shift in perspective has been reinforced by high-resolution profiling studies, including single-cell approaches, which reveal state-dependent keratinocyte programs associated with inflammatory skin disease and highlight that “keratinocyte” is not a single uniform entity but a collection of discrete, dynamically regulated states [10].

Baicalin, a flavone glycoside derived from Scutellaria baicalensis, has been reported to exhibit anti-inflammatory, immunomodulatory, and antioxidant activities across diverse experimental systems, and has been proposed as a candidate therapeutic agent for inflammatory disorders including skin inflammation [11, 12]. However, for AD specifically, key translational gaps remain: (i) the molecular targets of baicalin in the context of AD are incompletely defined; (ii) the cell type– and cell state–specific mechanisms (especially within the epidermis) remain unclear; (iii) and candidate targets often lack a tractable, testable chain of evidence linking transcriptomic prioritization to plausible binding or pathway modulation [13]. Addressing these gaps requires an approach that can simultaneously prioritize targets and localize their relevance within the cellular ecosystem of AD skin.

Given the cellular heterogeneity and stage-dependent biology of AD, resolving disease programs at the level of cell types and keratinocyte states is essential; however, transcriptomic signals alone do not readily translate into actionable therapeutic hypotheses. Single-cell RNA sequencing enables state-resolved localization of disease-associated programs within complex skin tissue [14]. Nominating druggable targets further requires integrating transcriptomic evidence with drug–target knowledge bases and disease gene resources, and prioritizing robust candidates using complementary feature-selection strategies such as LASSO and SVM-RFE [15]. Moreover, because transcriptome-based nomination does not directly address target engagement, structure-based modeling including docking and molecular dynamics can provide complementary in silico evidence by assessing whether candidate ligand–protein pairs are structurally compatible with binding in silico, thereby informing mechanistic hypotheses and improving experimental prioritization (Śledź and [16]).

Building on this framework, we aimed to prioritize candidate targets of baicalin in AD and to identify the most disease-responsive epidermal cell population and subpopulation using single-cell–resolved perturbation prioritization, followed by pathway-level characterization and structure-informed computational evaluation. Specifically, we combined pharmacogenomic target discovery and machine-learning-based prioritization with state-resolved single-cell analyses to generate a concise set of experimentally testable hypotheses linking baicalin, its putative target(s), and epidermal programs most closely associated with AD.

## Materials and methods

### Single-cell RNA sequencing data processing and quality control

Single-cell RNA sequencing (scRNA-seq) data were obtained from the Gene Expression Omnibus (GEO) database (accession number GSE153760) [17], comprising 8 atopic dermatitis (AD) donors and 7 healthy control donors (15 donors total) and processed in R (v4.4.1) using the Seurat package (v5.2.1) [18]. All samples were sequenced using the 10x Genomics Chromium platform on an Illumina HiSeq 3000 instrument. Prior to quality control, the dataset comprised approximately 15,000 cells in total. For each cell, quality-control metrics were computed, including the number of detected genes, the proportion of mitochondrial transcripts (genes matching the pattern *prefix* “MT-”), and the aggregate expression of hemoglobin genes (*HBA1, HBA2, HBB, HBD, HBE1, HBG1, HBG2, HBM, HBQ1, HBZ*) to flag potential erythrocyte contamination or cellular stress. Cells were retained if they contained 300–6,000 detected genes and fewer than 20% mitochondrial reads, resulting in 47,147 cells (AD: 28,768 cells; HC: 18,379 cells) retained for downstream analysis. Following quality-control filtering, expression matrices were normalized using Seurat’s LogNormalize method with a scale factor of 5,000 to account for differences in sequencing depth across cells. To mitigate potential batch effects across samples, we applied Harmony integration. Post-integration UMAP embeddings confirmed well-mixed sample distributions across major cell-type compartments, supporting the robustness of the data integration. Processed expression matrices, metadata, and dimensionality reduction embeddings are publicly available at GitHub: https://github.com/Vesemir-CN/AD-and-HC-single-cell-machine-learning-and-MD-dataset.

### Dimensionality reduction, integration, and clustering

Highly variable genes were identified using the variance-stabilizing transformation (VST) method, retaining the top 5,000 variable features for downstream analyses. Principal component analysis (PCA) was performed on scaled data. To mitigate batch effects across samples, Harmony integration was applied using *orig.ident* as the batch key. Clustering was performed on the Harmony-corrected space using *FindNeighbors* (dims = 1:30) and *FindClusters* (resolution = 0.5). Uniform Manifold Approximation and Projection (UMAP) was used for two-dimensional visualization based on the same 30 Harmony-corrected principal components.

### Cell type annotation and subset analysis

Cell clusters were annotated based on canonical marker genes and literature-curated cell-type signatures. For keratinocyte-focused analyses, keratinocyte cells were isolated and reanalyzed using the same preprocessing and clustering workflow, yielding 14 keratinocyte subclusters (Kcs1–Kcs14).

### Differential expression and pathway analysis

Differential expression analysis was conducted using the Wilcoxon rank-sum test implemented in the *presto* package. Genes meeting both log2FC > 0.5 and adjusted *P* < 0.05 were considered statistically significantly differentially expressed. For pathway-level interpretation, gene set enrichment analysis (GSEA) was performed using *clusterProfiler* with the MSigDB Hallmark (H) gene set collection. Pathways with FDR < 0.05 were considered significant, and enrichment magnitude were summarized using normalized enrichment scores (NES).

### Cell type prioritization using Augur

To prioritize cell types most responsive to CA2-associated stratification, Augur was applied as a cell-type prioritization framework [19]. Cells were stratified into CA2-high and CA2-low groups based on a median split of normalized *CA2* expression. Within each annotated cell type, Augur trained random forest classifiers to quantify the separability between CA2-high and CA2-low cells, reporting cross-validated area under the ROC curve (AUC). Cell types were ranked by AUC, with higher values indicating greater transcriptional separability under CA2 stratification.

### Machine learning-based consensus gene selection

To ensure biological independence and avoid pseudoreplication, single-cell expression profiles were aggregated into donor-level metacells using the hdWGCNA package (k and target metacell numbers auto-calculated based on group sizes), such that each metacell represents a transcriptionally coherent profile from a single donor. All machine learning analyses were performed on these metacells. We implemented a nested cross-validation framework via the easyScml pipeline (https://github.com/Vesemir-CN/easyscml). In the outer loop, donor-stratified 10-fold cross-validation (10 repeats) ensured that all metacells from the same donor were assigned exclusively to either the training or test set within each fold, preventing donor-level data leakage. The inner loop was reserved for hyperparameter optimization. Twenty machine learning algorithms were trained, and all 190 pairwise combinations (excluding self-pairing) were evaluated [20,21]; the combination heatmap diagonal displays single-algorithm performance for reference. Combinations achieving mean cross-validated AUC ≥ 0.8 were designated elite, and genes receiving votes from ≥ 50% of elite combinations were retained as consensus biomarkers. SHAP analysis was performed using an XGBoost classifier trained on the full metacell dataset to quantify directional feature contributions: positive SHAP values indicate contribution toward AD prediction; negative values toward HC. Point color in the beeswarm plot reflects normalized expression (blue: low; red: high). Visualizations were generated with ggplot2 (v3.4.0).

### Statistical analysis and visualization

All statistical analyses were performed in R, with multiple-testing correction applied using the Benjamini–Hochberg false discovery rate procedure where applicable. Visualization was performed using *ggplot2*, *pheatmap*, and custom plotting functions. UMAP projections were generated using Seurat, and pathway results were visualized using *clusterProfiler* functions.

### Cell state distance analysis using scDist

To quantify transcriptomic distances between CA2-stratified states while accounting for sample-level variation, scDist was applied to the keratinocyte subset [22]. CA2-high versus CA2-low status (*newgroup*) was modeled as a fixed effect, and sample identity (orig.ident) was included as a random effect. Keratinocyte subclusters (Kcs1–Kcs14) were used as the cell-state labels. Distances were computed in a reduced-dimensional space (*d* = 20 principal components), yielding a quantitative estimate of CA2-associated perturbation magnitude across keratinocyte subpopulations.

scHSC single-cell fine clustering

Single-cell Hierarchical Spectral Clustering (scHSC) was used as an additional unsupervised fine-clustering strategy [23]. Briefly, scHSC constructs a k-nearest neighbor (*k*-NN) graph from normalized expression profiles, performs spectral embedding, and applies hierarchical clustering in the embedded space. The optimal number of clusters was determined by the silhouette coefficient. Analyses were performed in R (v4.4.2) using the *scHSC* package with default parameters unless otherwise specified.

### Molecular docking analysis

Molecular docking was conducted to evaluate the binding feasibility and predicted interaction mode of baicalin with carbonic anhydrase II (CA2) [24]. The CA2 crystal structure was retrieved from the RCSB Protein Data Bank (PDB ID: 1LUG) and prepared by removing crystallographic water molecules and non-essential heteroatoms. The baicalin structure was obtained from PubChem and converted to a 3D conformer with energy minimization in Chem3D (MM2 force field), followed by format conversion using AutoDockTools (v1.5.7). Docking was performed with AutoDock Vina using a grid box (48.8 × 46.7 × 56.9 Å) centered on the CA2 active site. Key parameters were set as follows: exhaustiveness = 10, energy_range = 4 kcal/mol, and n_modes = 10. The top-ranked binding pose was selected for subsequent simulation, and protein–ligand interactions were inspected in MOE.

### Molecular dynamics simulations

Molecular dynamics (MD) simulations were performed using GROMACS (v2025.3) to assess the conformational stability of the baicalin–CA2 complex in explicit solvent. The AMBER14SB force field was applied to the protein, and the system was solvated with TIP3P water molecules in a periodic boundary condition simulation box. Ligand topology and force field parameters were generated using Sobtop (v1.0-dev5). The system was charge-neutralized and supplemented with 150 mM NaCl to mimic physiological ionic conditions. Following energy minimization, the system was equilibrated sequentially under NVT and NPT ensembles, followed by a 100 ns production run with a 2 fs integration time step. Long-range electrostatics interactions were treated using the particle mesh Ewald (PME) method, and bonds involving hydrogen atoms were constrained using the LINCS algorithm. Production trajectories were analyzed to compute root mean square deviation (RMSD), radius of gyration (Rg), root mean square fluctuation (RMSF), solvent-accessible surface area (SASA), and the number of protein–ligand hydrogen bonds.

### Binding free energy estimation (MM-PBSA)

Binding free energy of the baicalin–CA2 complex was estimated using the molecular mechanics Poisson–Boltzmann surface area (MM-PBSA) method applied to snapshots extracted from MD trajectories [25]. The total binding free energy was decomposed into molecular mechanics terms (van der Waals and electrostatic interactions) and solvation free energy terms (polar and non-polar components). Per-residue energy decomposition was additionally performed to identify residues contributing most strongly to the estimated binding free energy.

### Free energy landscape (FEL) analysis

To characterize the dominant conformational states sampled during MD simulation, free energy landscapes (FELs) were constructed using backbone RMSD and radius of gyration (*Rg*) as reaction coordinates [26]. Free energy surfaces were derived from the Boltzmann-weighted probability distributions of these coordinates to visualize major low-energy basins and conformational preferences of the baicalin–CA2 complex.

## Results

**Fig 1:**
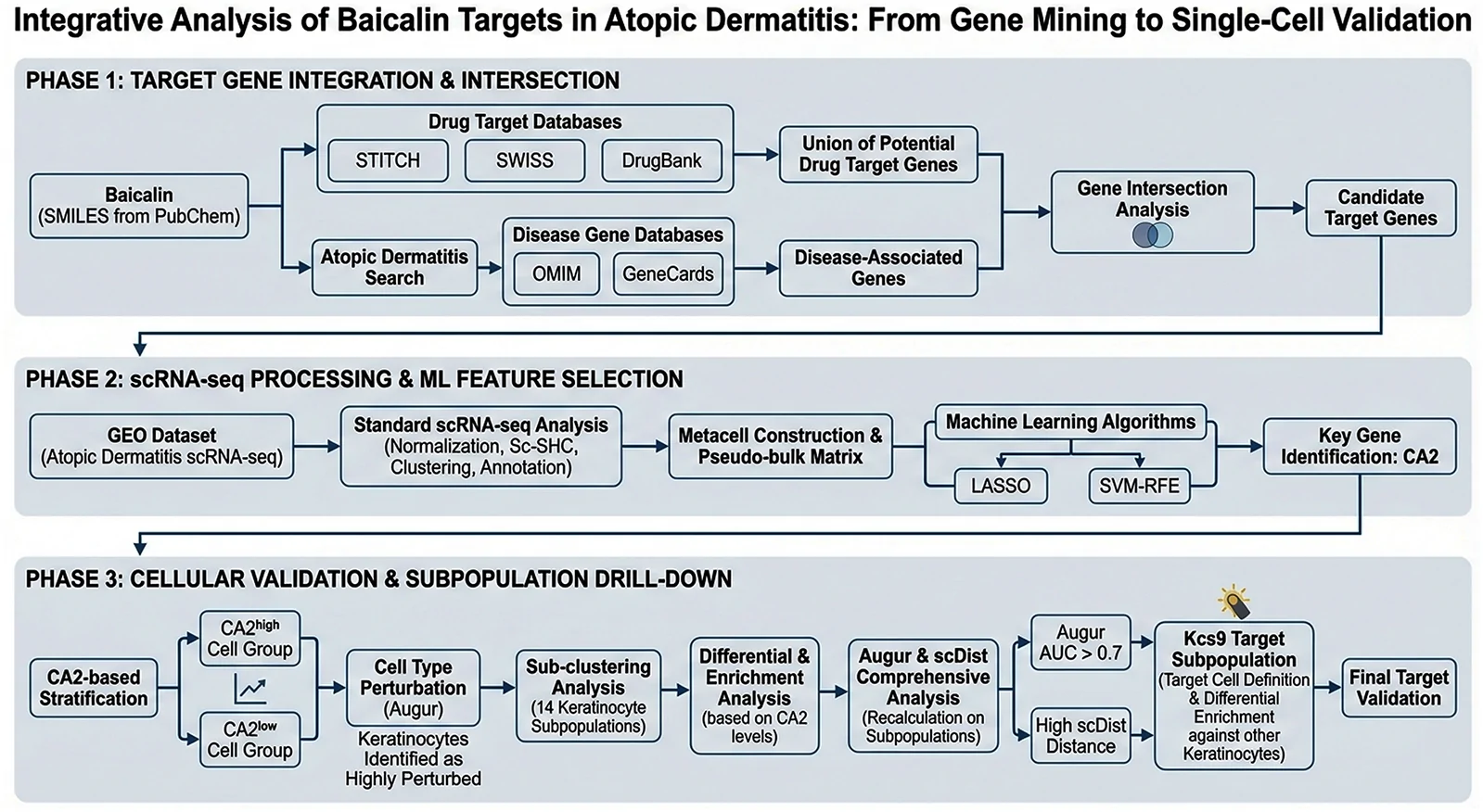
Integrated pharmacogenomics and single-cell workflow for atopic dermatitis. Schematic overview of the three-phase analysis pipeline. Phase 1 (Target Gene Integration & Intersection): Baicalin drug targets were retrieved via PubChem SMILES and queried against STITCH, Swiss Target Prediction, and DrugBank to generate a union of potential target genes; AD-associated genes were independently mined from OMIM and GeneCards. The two sets were intersected to derive candidate target genes. Phase 2 (scRNA-seq Processing & ML Feature Selection): Public AD scRNA-seq data (GEO) were processed through normalization, ScSHC–based clustering, and cell type annotation, followed by metacell construction and pseudo-bulk matrix generation. Candidate genes were prioritized by LASSO and SVM-RFE, identifying CA2 as the top-ranked computational candidate. Phase 3 (Cellular Validation & Subpopulation Drill-Down): Cells were stratified into CA2-high and CA2-low groups; Augur separability analysis revealed keratinocytes as the most transcriptionally separable cell type under CA2 stratification. Keratinocytes were sub-clustered into 14 subpopulations (Kcs1–Kcs14) and subjected to differential and enrichment analyses based on CA2 levels. Augur and scDist analyses recalculated on subpopulations nominated Kcs9 via dual criteria (Augur AUC > 0.7 and high scDist distance); Kcs9 was further characterized by differential enrichment against other keratinocyte subpopulations, providing a computationally prioritized candidate state for experimental follow-up..

**Fig 2:**
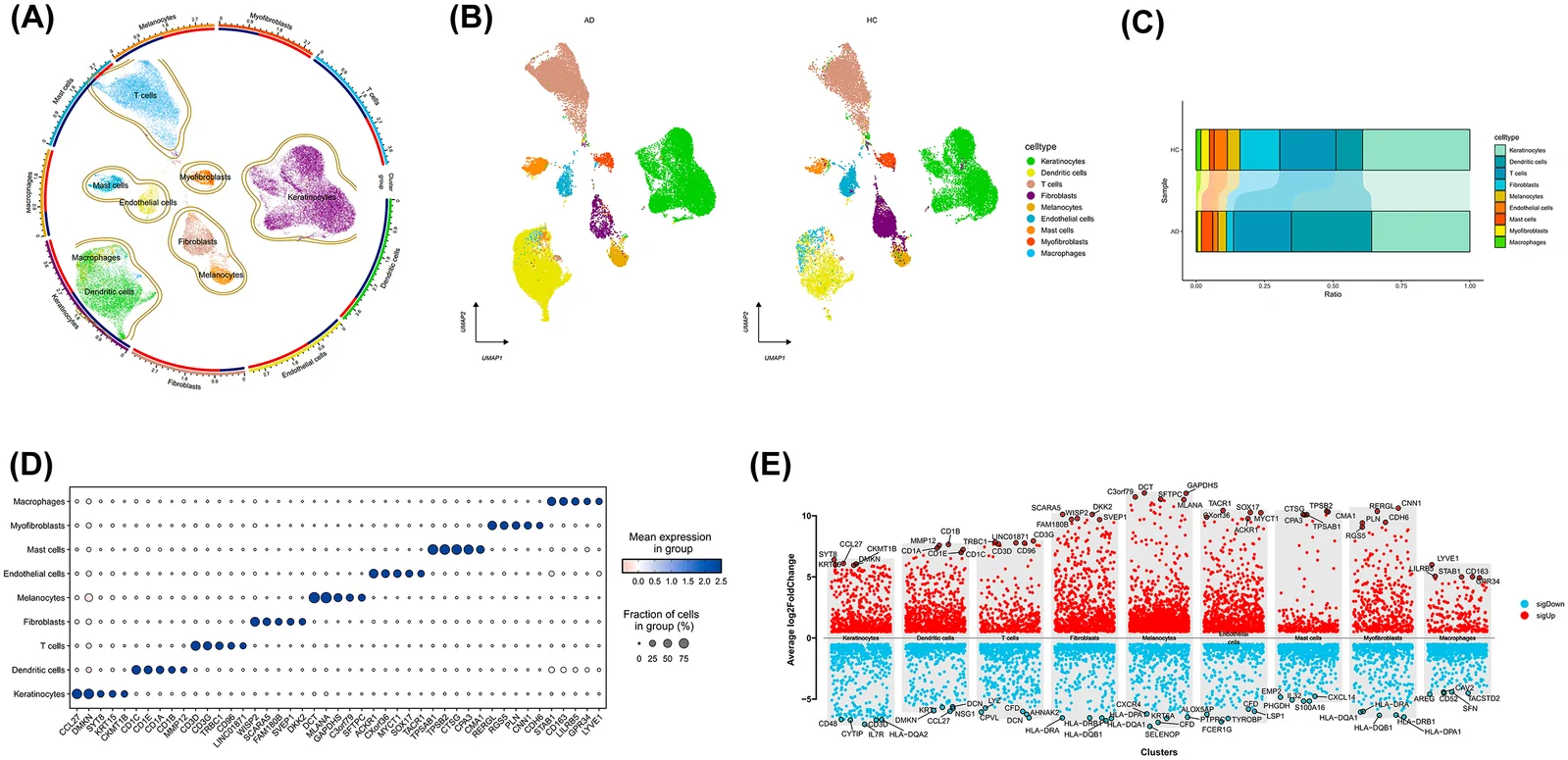
Single-cell atlas construction and cell-type annotation of AD skin. (A) Global embedding of all cells showing major cell types in the integrated AD skin atlas. (B) UMAPs split by group (AD and HC) with cells colored by annotated cell type. (C) Stacked bar chart summarizes cell-type composition by group. (D) Dot plot of canonical marker genes across major cell types. (E) Differential expression summary by cell type (average log2 fold-change), highlighting significantly upregulated and downregulated genes across clusters.

**Fig 3:**
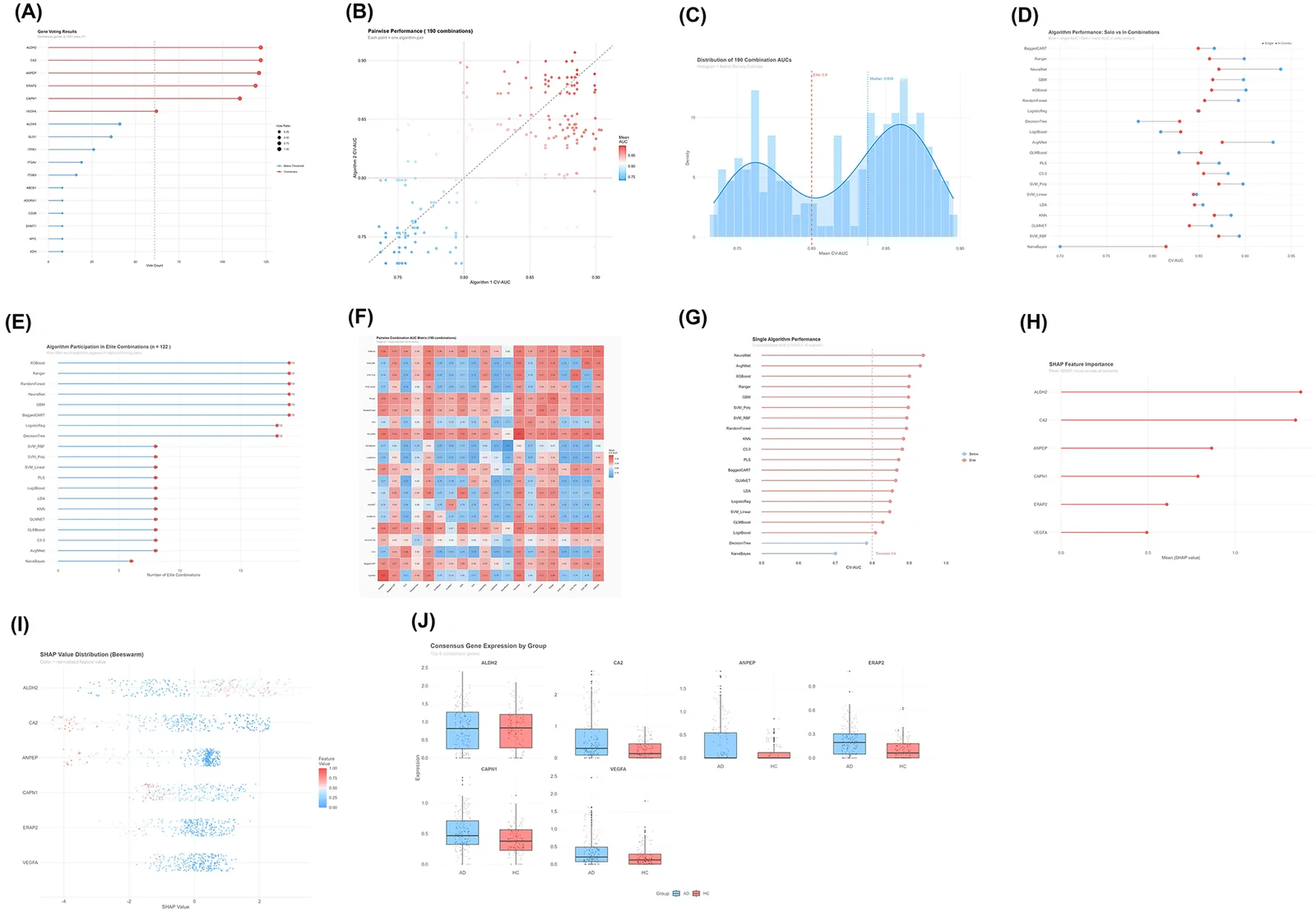
Machine learning consensus screening and SHAP interpretability for biomarker discovery. A) Cross-validated AUC values (mean ± SD) for 20 individual machine learning algorithms. Dashed line indicates the elite threshold (AUC = 0.8). (B) Scatter plot of pairwise algorithm performance, with x- and y-axes representing the cross-validated AUC of each individual algorithm in the pair, colored by mean combination AUC. (C) Density distribution of combination AUCs with median (solid line) and elite threshold (dashed line) indicated. (D) Dumbbell plot comparing single-algorithm AUC (blue) versus mean AUC across elite combinations in which each algorithm participated (red). (E) Algorithm participation frequency, expressed as the number of elite combinations in which each algorithm participated. (F) Heatmap of pairwise combination AUCs for all 190 algorithm pairs (excluding self-pairing). Diagonal displays single-algorithm performance for reference; color scale from blue to red indicates increasing AUC. (G) Voting results of genes across elite combinations. Red bars denote consensus genes passing the 50% vote threshold; green bars indicate non-consensus genes. (H) Global SHAP feature importance ranking based on mean absolute SHAP values derived from the XGBoost classifier. (I) SHAP beeswarm summary plot derived from the XGBoost classifier. Points are colored by normalized feature expression (blue: low; red: high); positive SHAP values favor AD prediction, negative values favor HC prediction. (J) Normalized expression distribution of six consensus genes across AD and HC groups. Significance was determined by two-sided Wilcoxon rank-sum tests (*p < 0.05, **p < 0.01, ***p < 0.001).

**Fig 4:**
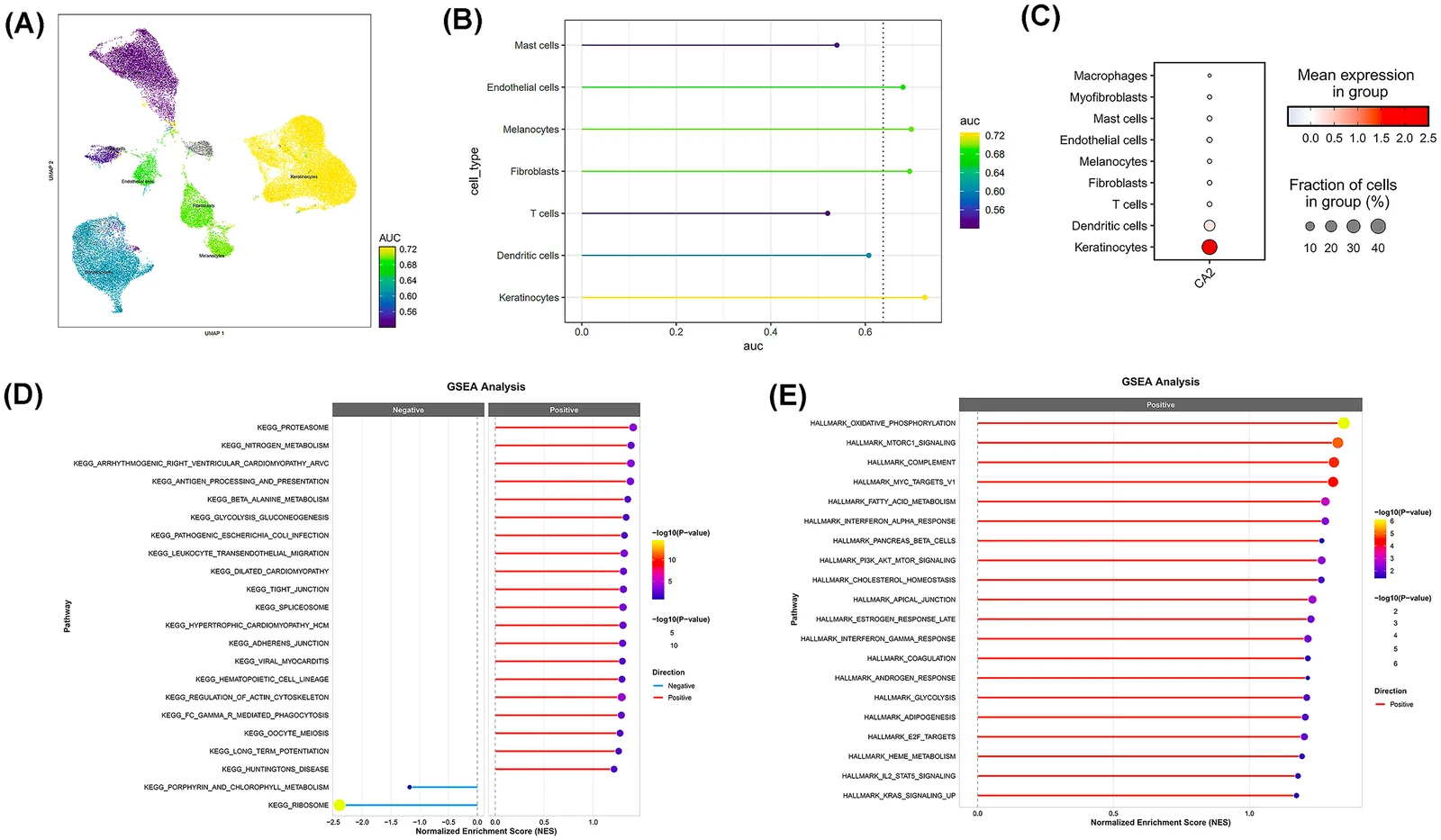
CA2 expression distribution across cell types and functional enrichment associated with CA2 stratification. (A) Augur AUC projected onto the integrated skin scRNA-seq UMAP, quantifying within–cell type transcriptomic separability between CA2-high vs CA2-low cells (higher AUC indicates stronger separability). (B) Augur AUC summarized across major cell types, ranking cell types by sensitivity to CA2 stratification. (C) Dot plot showing CA2 expression across major cell types; dot size indicates the fraction of expressing cells and color indicates mean expression. (D) KEGG gene set enrichment analysis (GSEA) comparing (CA2-high versus CA2-low cells), shown as normalized enrichment score (NES) with enrichment direction (red, positive; blue, negative) and significance (−log10 P value). (E) HALLMARK GSEA comparing (CA2-high versus CA2-low cells), shown as NES with enrichment direction (red, positive) and significance (−log10 P value).

**Fig 5:**
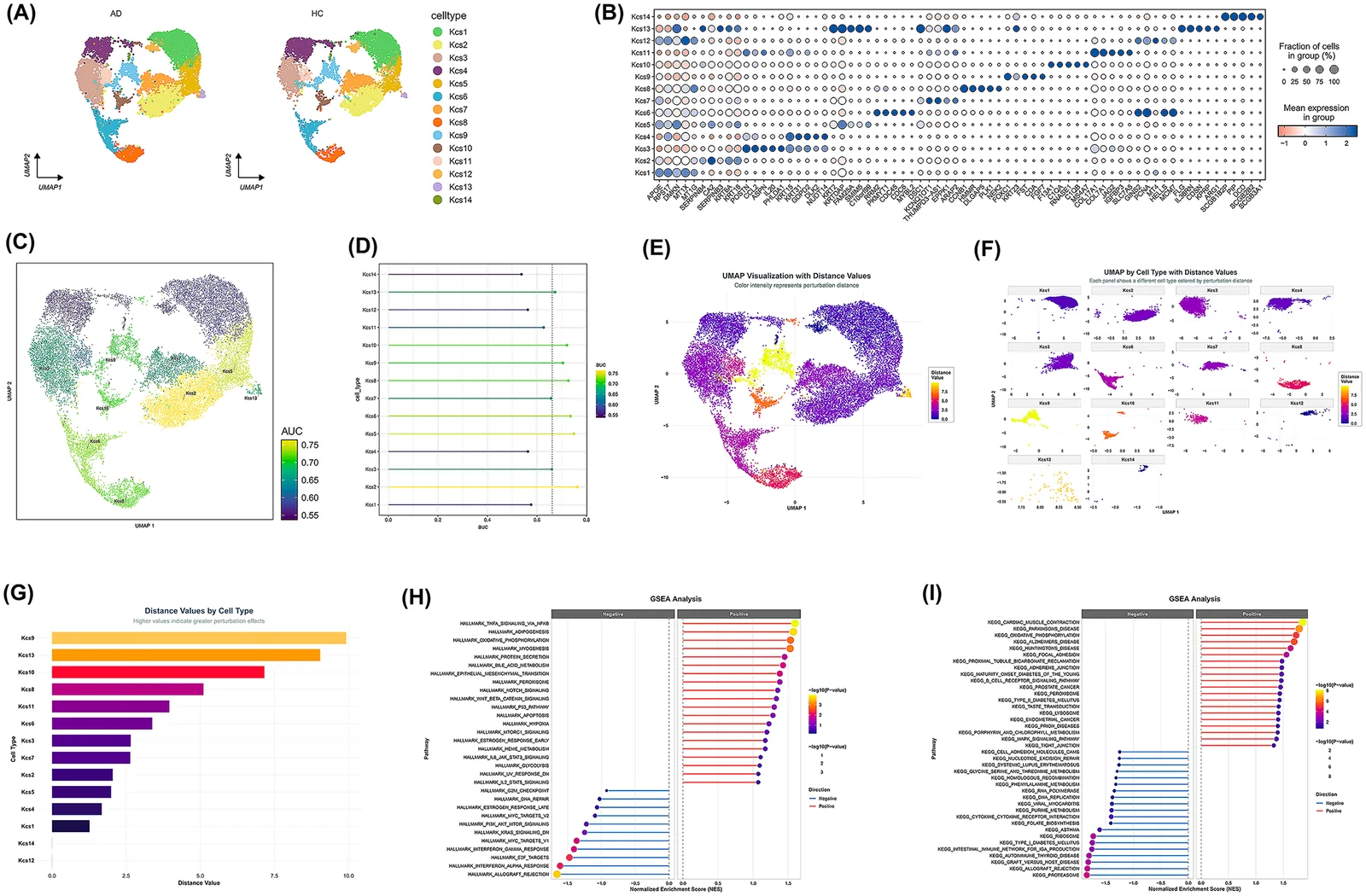
Subpopulation-resolved Augur and scDIST analysis nominates Kcs9 as the most CA2-associated transcriptionally divergent keratinocyte state. (A) Keratinocyte subset UMAPs split by group (AD and HC), showing 14 keratinocyte subclusters (Kcs1–Kcs14) to assess cross-group coverage and subcluster structure. (B) Dot plot of marker genes across Kcs1–Kcs14; dot size indicates the fraction of expressing cells and color indicates mean expression. (C) Keratinocyte UMAP colored by Augur AUC across all keratinocyte subclusters, where color intensity represents the magnitude of transcriptomic separability associated with CA2 expression status. (D) Augur AUC summarized by keratinocyte subcluster (Kcs1–Kcs14), ranking subclusters by CA2-stratified separability (higher AUC indicates stronger transcriptomic separability). (E) Keratinocyte UMAP colored by scDist-estimated transcriptomic distance, where color intensity represents the magnitude of CA2-associated transcriptomic distance in multivariate expression space. (F) Faceted UMAPs by keratinocyte subcluster with scDist-estimated transcriptomic distance overlaid, highlighting subcluster-specific transcriptomic distance “hotspots”. (G) Bar plot ranking scDist-estimated transcriptomic distance across Kcs1–Kcs14 (higher distance indicates stronger CA2-associated perturbation), nominating Kcs9 as the top-ranked transcriptionally divergent keratinocyte state. (H) HALLMARK GSEA for CA2-high vs CA2-low within Kcs9, reporting normalized enrichment scores (NES) and significance. (I) KEGG GSEA for CA2-high vs CA2-low within Kcs9, reporting normalized enrichment scores (NES) and significance.

**Fig 6:**
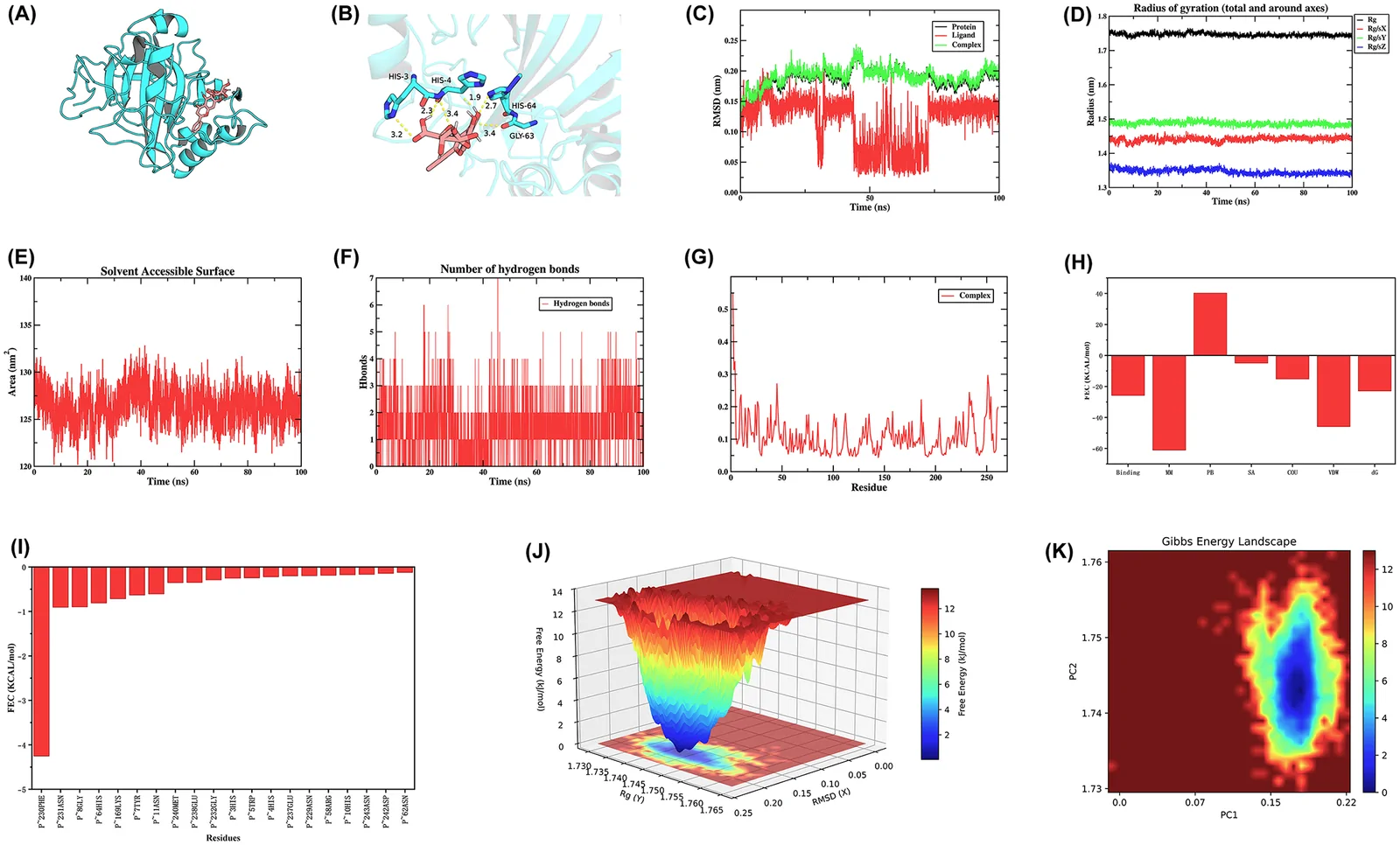
Molecular docking and molecular dynamics simulations support binding feasibility of baicalin to CA2. (A) Overall docking pose of baicalin in the CA2 structure; protein is shown as cartoon and baicalin as sticks to illustrate the predicted binding location within a defined pocket. (B) Zoomed-in view of the docking site highlighting key interacting residues and representative polar contacts (distances in Å), indicating a plausible hydrogen-bonding interaction network. (C) RMSD trajectories over 100 ns for protein, ligand, and the complex, assessing conformational stability and equilibration behavior during MD simulation. (D) Radius of gyration (Rg) over 100 ns (total and axis-resolved components) to evaluate global compactness of the protein in the bound state. (E) Solvent accessible surface area (SASA) over 100 ns to quantify changes in solvent exposure of the complex during MD simulation. (F) Number of protein–ligand hydrogen bonds over time to summarize persistence and dynamics of polar interactions across the trajectory. (G) Residue-wise RMSF profile of the complex to characterize local flexibility and identify regions with higher conformational fluctuations. (H) MM-PBSA binding free energy components for the baicalin–CA2 complex, reporting the contributions of molecular mechanics terms (e.g., van der Waals and electrostatics) and solvation terms (polar and nonpolar), together with the estimated overall binding free energy. (I) Per-residue free energy decomposition (MM-PBSA) showing residues with the strongest favorable contributions (“hotspots”) to the estimated binding free energy. (J) Free energy landscape (FEL) of the baicalin–CA2 complex derived from the MD trajectory, visualized as a 3D surface plot with RMSD and Rg as reaction coordinates, illustrating dominant low-energy conformational basins. (K) Two-dimensional Gibbs free energy landscape projected onto the first two principal components (PC1 and PC2) from principal component analysis (PCA) of the MD trajectory, highlighting the predominant low-energy conformational state sampled during simulation.

We developed an integrated, single-cell–resolved framework to prioritize putative targets of baicalin in atopic dermatitis (AD) and to computationally identify the epidermal cell populations most responsive to CA2 stratification (Fig 1). As outlined in Fig 1, the workflow links drug–target inference with AD-associated gene resources and skin scRNA-seq data, and then refines candidates using machine-learning–based prioritization. The subsequent results present the prioritized target and its cell-state specificity, followed by pathway-level interpretation and structure-informed feasibility assessment (Figs 2–6).

### A quality-controlled, integrated single-cell atlas reveals separable cell communities in AD and control skin

To provide a robust single-cell reference for downstream target prioritization and cell-population localization (Fig 1), we constructed an integrated AD–HC skin atlas through harmonized preprocessing, integration, and unsupervised clustering. The resulting atlas, visualized as a circular embedding in which each arc segment represents a major cell-type compartment, displayed well-separated cellular communities with distinct boundaries across nine annotated lineages, supporting adequate global resolvability of major skin cell populations (Fig 2A). With this atlas structure in place, we next evaluated cross-group comparability within the same reference space. AD and HC cells occupied the same major cellular compartments on the shared embedding, indicating that subsequent AD–HC contrasts can be performed under a consistent cellular framework rather than being confounded by missing lineages in one group (Fig 2B). At the level of tissue cellular ecology, cell-type composition differed markedly between AD and HC: keratinocytes accounted for a substantially larger proportion in AD relative to HC, whereas immune cell populations—including T cells and dendritic cells—showed comparatively higher representation in HC, consistent with the epidermal hyperplasia and immune infiltration patterns characteristic of AD-associated tissue remodeling (Fig 2C). Annotation reliability was then assessed using canonical marker expression patterns. Representative lineage markers exhibited cell-type–specific enrichment across clusters—exemplified by epidermal keratinocyte markers (e.g., KRT15, DMKN), immune markers for T cells (e.g., CD3D, TRBC1), and myeloid/mast-cell–associated markers (e.g., CD1C; TPSAB1/CPA3)—supporting reliable downstream cell-type labeling based on canonical marker concordance (Fig 2D). Finally, we examined whether group-associated transcriptional differences were cell-type interpretable, rather than reflecting diffuse noise. Comparing AD to HC within each annotated cluster, distinct cell-type–specific differential expression patterns emerged consistent with expected biological roles: keratinocyte clusters exhibited upregulation of structural epidermal genes (e.g., KRT15, DMKN) alongside downregulation of differentiation-associated transcripts, while immune compartments—including T cells, dendritic cells, and mast cells—displayed upregulation of markers associated with activation and inflammatory state (e.g., CD3G, TRBC1, TPSAB1). These cell-type–interpretable transcriptional differences, rather than diffuse noise, provide a structured computational framework for subsequent CA2-associated feature prioritization and stratification analyses (Fig 2E).

### Machine learning‑based consensus screening and SHAP interpretation prioritize a panel of robust AD‑associated genes

To prioritize candidate biomarkers with high cross-validated discriminative performance, we benchmarked 20 diverse machine learning algorithms on donor-level metacells under a nested, donor-stratified cross-validation framework. Single-algorithm evaluation revealed substantial performance heterogeneity across methods, with a subset of algorithms exceeding the predefined elite threshold of AUC = 0.8 (Fig 3A). To capture complementary algorithmic strengths, we systematically constructed all 190 pairwise algorithm combinations and assessed their combinatorial performance. Scatter plot visualization of pairwise AUCs demonstrated that high-performing combinations clustered distinctly from lower-performing pairs (Fig 3B), and density distribution analysis confirmed that the majority of elite combinations were concentrated above the AUC = 0.8 threshold, with the median combination AUC exceeding that of most individual algorithms (Fig 3C). Dumbbell plot comparison further illustrated that participation in elite combinations consistently elevated mean AUC relative to single-algorithm baselines for most methods (Fig 3D), while algorithm participation frequency analysis identified a core set of algorithms that disproportionately contributed to elite combinations, suggesting their superior generalizability in this dataset (Fig 3E). The full combination performance landscape was summarized in a pairwise AUC heatmap, which revealed structured blocks of high-performing algorithm pairs and confirmed the robustness of the combinatorial evaluation strategy (Fig 3F). Gene-level voting across all elite combinations then identified six consensus biomarkers surpassing the 50% vote threshold, with CA2 receiving the highest vote frequency among all candidates (Fig 3G). To interpret the predictive contribution of each consensus gene, SHAP analysis was performed using an XGBoost classifier; global feature importance ranking confirmed CA2 as the dominant predictive feature by mean absolute SHAP value (Fig 3H), and the beeswarm summary plot demonstrated that elevated CA2 expression was consistently associated with positive SHAP values, indicating a robust directional contribution toward AD prediction across individual metacells (Fig 3I). Finally, expression distribution analysis across AD and HC groups validated that all six consensus genes exhibited statistically significant differential expression, with CA2 showing the most pronounced and consistent upregulation in AD (Fig 3J; Wilcoxon rank-sum test, p < 0.001).

### CA2 stratification prioritizes keratinocytes as the most transcriptionally separable cell type and associates with broad pathway enrichment signals

Building on the nomination of CA2 and its disease-associated expression support (Fig 3), we next asked which major cell types exhibit the most substantial transcriptomic separability under CA2 stratification (CA2-high vs CA2-low). To quantify this in a cell-type–aware manner, we applied Augur, which measures within–cell type separability using an AUC-based prioritization score.

Projecting Augur AUC onto the integrated UMAP revealed marked spatial heterogeneity in separability across the atlas, with the strongest signal mapping to the keratinocyte-dominated region (Fig 4A). Consistently, cell type–level summaries showed that keratinocytes ranked highest in AUC, suggesting that keratinocytes exhibited the strongest CA2-stratified transcriptional separability among all major cell types, as ranked by Augur AUC (Fig 4B).

To contextualize this prioritization, we examined the distribution of CA2 expression across major cell types. A dot-plot overview showed that keratinocytes display the highest CA2 signal in terms of both mean expression and the fraction of expressing cells, consistent with their top-ranked separability under CA2-high vs CA2-low stratification (Fig 4C).

We then asked what pathway-level enrichment patterns accompany CA2 stratification at the pathway level. Gene set enrichment analyses identified coordinated shifts across multiple functional modules rather than an isolated pathway change. KEGG GSEA highlighted significant enrichment involving metabolic reprogramming, epithelial junction and barrier remodeling, and proliferative quiescence (Fig 4D) (S1 Table). In parallel, HALLMARK GSEA implicated broad programs spanning energy metabolism and signaling/inflammatory axes (Fig 4E) (S1 Table). Together, these results position keratinocytes as the most CA2-sensitive major cell type in terms of transcriptomic separability and suggest that CA2 stratification is associated with multi-program reconfiguration across curated pathway resources.

### Subpopulation-resolved prioritization identifies Kcs9 as the most CA2-associated perturbed keratinocyte state

To localize CA2-associated transcriptional differences within the keratinocyte compartment, we constructed a keratinocyte sub-atlas comprising 14 subclusters (Kcs1–Kcs14). Visualization of the keratinocyte embedding split by group (AD vs HC) showed that major subclusters were represented across conditions, supporting adequate cross-group coverage for downstream CA2-based stratification analyses (Fig 5A). Marker-gene dot plots further summarized the transcriptional features distinguishing Kcs states, providing a reference map for interpreting where CA2-linked signals concentrate within the keratinocyte landscape (Fig 5B).

We first quantified CA2-stratified separability within each keratinocyte state using Augur. Projecting Augur AUC onto the keratinocyte UMAP revealed substantial heterogeneity across the sub-atlas, suggesting that separability between CA2-high vs CA2-low is subcluster-dependent rather than uniformly distributed across keratinocytes (Fig 5C). Consistently, subcluster-level AUC summaries ranked keratinocyte states by CA2-associated separability, highlighting a subset of high-sensitivity states and nominating Kcs9 among the top prioritized populations (Fig 5D).

To quantify perturbation magnitude in multivariate expression space, we next applied scDist. Mapping scDist distance onto the keratinocyte UMAP showed spatially concentrated regions of elevated distance, consistent with discrete subpopulation-associated shifts rather than diffuse cell-to-cell noise (Fig 5E). Faceted UMAPs further demonstrated that these distance “hotspots” localize to specific keratinocyte subclusters (Fig 5F). When summarized across Kcs1–Kcs14, scDist distances robustly ranked keratinocyte states by CA2-associated transcriptomic distance, with Kcs9 exhibiting the largest distance estimate, thereby nominating it as the most CA2-associated perturbed keratinocyte state (Fig 5G).

Finally, to interpret the biological programs accompanying CA2 stratification within the prioritized state, we performed pathway enrichment within Kcs9. HALLMARK GSEA showed enrichment signals across metabolic and signaling/stress-response programs, including modules related to oxidative phosphorylation and pathway-level signaling signatures (Fig 5H). In parallel, KEGG GSEA highlighted enrichment patterns involving cellular adhesion/junctional organization and multiple metabolism- and immune-related pathways, consistent with the notion that CA2 stratification in Kcs9 is associated with enrichment patterns spanning multiple gene-set modules across curated resources (Fig 5I) (S1 Table).

### Structure-based docking and molecular dynamics provide computational support for binding feasibility of baicalin to CA2

To provide orthogonal, structure-based support for CA2 as a plausible baicalin-interacting candidate, we performed molecular docking followed by 100-ns molecular dynamics (MD) simulations and post hoc binding-energy analyses (Fig 6A–K). Docking yielded a coherent binding pose in which baicalin occupied a defined pocket on CA2 (Fig 6A). Local interaction inspection suggested a hydrogen-bond–supported polar contact network involving His3, His4, Gly63, and His64, with interaction distances within a typical hydrogen-bonding range (approximately 1.9–3.4 Å), consistent with a geometrically plausible docked pose (Fig 6B). We then assessed complex stability during MD simulation. RMSD trajectories indicated an initial equilibration phase followed by a plateau-like regime; the protein RMSD remained stable throughout the trajectory, fluctuating around ~0.17–0.18 nm, while the complex RMSD fluctuated around ~0.18–0.20 nm without a pronounced drift, whereas the ligand RMSD exhibited larger fluctuations throughout the trajectory, consistent with greater conformational flexibility of the free ligand relative to the bound protein (Fig 6C). In parallel, the radius of gyration (Rg) remained largely stable at approximately 1.75–1.80 nm throughout the 100-ns trajectory, with axis-resolved components (Rg/sX ~ 1.44 nm, Rg/sY ~ 1.49 nm, Rg/sZ ~ 1.35 nm) similarly showing minimal drift, collectively suggesting maintenance of global compactness of CA2 during the simulation trajectory (Fig 6D). Solvent accessible surface area (SASA) similarly showed no marked shift across the trajectory, fluctuating around a mean of approximately ~127 nm² within a range of ~121–133 nm², suggesting that no large-scale changes in protein surface exposure were observed during the simulation (Fig 6E). Hydrogen-bond analysis further showed persistent but dynamic protein–ligand hydrogen bonding over time (typically ~2–3 hydrogen bonds, ranging from 0 to 7), consistent with persistent yet dynamic hydrogen-bonding patterns during simulation (Fig 6F). RMSF remained low for most residues (predominantly <0.20 nm), with the exception of N-terminal residues (~residues 1–5) exhibiting the highest fluctuations (~0.54 nm) and C-terminal residues (~residues 220–265) showing moderately elevated fluctuations (peaking at ~0.30 nm), suggesting low overall residue-level fluctuation with localized flexibility at both terminal regions (Fig 6G). To quantify binding energetics, MM-PBSA yielded an estimated binding free energy for the baicalin–CA2 complex (ΔG_bind ≈ −22.082 kcal/mol), suggesting a potentially favorable interaction within the limitations of this approximate method, with van der Waals interactions (VDW ≈ −46 kcal/mol) representing the dominant favorable contribution, supplemented by electrostatic interactions (COU ≈ −15 kcal/mol), collectively offset by unfavorable polar solvation (PB ≈ +40 kcal/mol), while nonpolar solvation (SA ≈ −3 kcal/mol) contributed modestly favorably (Fig 6H). Per-residue free-energy decomposition further highlighted a subset of pocket residues with stronger favorable contributions, with Phe230 and Asn231 exhibiting the largest individual contributions, nominating these residues as computationally predicted interaction hotspots that may contribute disproportionately to the estimated binding free energy (Fig 6I). FEL analysis revealed a single dominant low-energy basin with a well-defined funnel-shaped profile (centered at Rg ~ 1.750 nm and RMSD ~0.05–0.10 nm, free energy ~0 kJ/mol), surrounded by high-energy regions reaching ~13–14 kJ/mol, suggesting the presence of a dominant low-energy conformational basin sampled during the trajectory (Fig 6J). Two-dimensional Gibbs free energy landscape projected onto PC1 and PC2 from principal component analysis (PCA) further confirmed a single predominant low-energy conformational state sampled during simulation, consistent with a stable and well-defined bound-state ensemble (Fig 6K). Collectively, these docking and MD results provide consistent in silico observations supporting the computational plausibility of baicalin binding to CA2 and motivate downstream experimental investigation of this predicted interaction.

## Discussion

In this study, we integrated pharmacogenomic target mining, machine-learning prioritization, and single-cell state–resolved perturbation analyses to identify actionable epidermal targets and responsive keratinocyte states in atopic dermatitis (AD). Across multiple, methodologically distinct lines of computational evidence, CA2 emerged as a convergent candidate, supported by drug–target/disease-gene intersection, orthogonal feature-selection approaches (LASSO and SVM-RFE), and disease-associated expression patterns. Leveraging single-cell resolution, we further localized the CA2-associated signal to the epidermal compartment, where keratinocytes exhibited the strongest CA2-stratified separability and transcriptional divergence. Subclustering refined this observation to a prioritized keratinocyte state (Kcs9), consistently highlighted by both Augur AUC–based separability and scDist-estimated transcriptomic distance. At the pathway level, CA2 stratification was associated with enrichment signals spanning multiple pathway modules rather than a single isolated gene set, encompassing epithelial junction and structural remodeling together with metabolic, epithelial junction and barrier remodeling-related programs. Finally, structure-based modeling offered an orthogonal structural plausibility layer by supporting a feasible baicalin–CA2 interaction in silico. Importantly, the single-cell analyses establish CA2 as a disease-relevant axis localized to specific keratinocyte states, whereas the structure-based results serve as a feasibility argument for pharmacological engagement—not evidence of in vivo efficacy. Together, these results motivate a testable target–state hypothesis that links an actionable molecule–target pair to a specific disease-relevant keratinocyte state.

### Mechanistic interpretation: A coupled “state-network” response in keratinocytes

A useful way to interpret our findings is through a state-network hypothesis: in the inflammatory microenvironment of AD, keratinocytes may transition into adaptive states characterized by concurrent structural remodeling and metabolic/proteostatic stress. In this framework, enrichment of junctional/cytoskeletal and adhesion-related programs can be viewed as a response to barrier disruption and repeated injury–repair cycles, while concurrent shifts in oxidative phosphorylation, glycolysis, and protein-processing pathways are consistent with increased energetic demand and cellular stress management required to sustain these remodeling programs.

Within such a coupled network, CA2—an enzyme central to pH and bicarbonate/ion homeostasis—may either functionally contribute to, or serve as a transcriptional marker of, keratinocyte adaptation to inflammatory stress. Critically, independent experimental evidence supports the disease relevance of CA2 in AD epidermis: Kamsteeg et al. [27] demonstrated that CA2 mRNA and protein are significantly upregulated in lesional AD skin compared with both psoriatic lesions and healthy controls, with expression localized predominantly to suprabasal, differentiated keratinocytes. Importantly, this upregulation was shown to be driven by Th2 cytokines (IL-4 and IL-13) rather than Th1 stimulation, directly linking CA2 induction to the canonical AD inflammatory microenvironment rather than reflecting a cell-autonomous, genetically programmed difference. This experimentally established expression pattern is consistent with the CA2-associated transcriptomic signal identified in our single-cell analyses, and provides external biological plausibility for the computational nominations reported here. pH and ion handling are tightly linked to epidermal differentiation, stratum corneum enzymatic activity, and barrier function [28,29]; elevated skin surface pH has been reported in AD patients, and alkalinization of the stratum corneum has been shown to impair barrier homeostasis [27], further contextualizing CA2 dysregulation within the barrier–inflammation axis characteristic of AD. Thus, the co-enrichment of modules of metabolic reprogramming, epithelial junction and barrier remodeling, and proliferative quiescence observed in CA2-stratified keratinocytes is not simply a list of unrelated pathways, but a coherent signature of keratinocytes operating under combined barrier-repair demand and homeostatic pressure.

Notably, the prioritization of Kcs9 by both separability (Augur) and transcriptomic distance (scDist) suggests that this subpopulation represents a high-sensitivity keratinocyte state in which CA2 stratification aligns with coordinated remodeling and stress-adaptation programs. Conceptually, Kcs9-like cells may therefore constitute a ‘response-prone’ epidermal node where barrier-associated structural programs and homeostatic stress programs show concurrent enrichment signals, making them informative for disease stratification and mechanistic testing. The biological identity of Kcs9 is further informed by the convergence of its five cluster-defining marker genes—FOXC1, KRT23, FST, CDA, and FGF7—with its GSEA enrichment architecture. FST, a secreted BMP/Activin antagonist documented in suprabasal keratinocyte compartments of inflamed skin [30], aligns with positive enrichment of TIGHT_JUNCTION, ADHERENS_JUNCTION, NOTCH, and WNT_BETA_CATENIN signals, positioning Kcs9 within a barrier-competent suprabasal differentiation context; notably, NOTCH/WNT attenuation drives keratinocyte fate divergence toward inflammatory states, suggesting that their sustained enrichment in Kcs9 reflects active barrier maintenance rather than default differentiation. KRT23 co-expression further supports a spinous-layer identity consistent with KRT1/KRT10-anchored coordinates in single-cell atlases. FGF7-associated enrichment of PROTEIN_SECRETION, MTORC1_SIGNALING, and MAPK—alongside negative enrichment of CYTOKINE_CYTOKINE_RECEPTOR_INTERACTION—indicates growth factor–biased rather than cytokine-amplifying secretory output, mechanistically distinct from the LAMB3–CD44 and integrin-dominated pro-inflammatory KC communication programs in AD lesional skin. CDA-linked dual enrichment of OXIDATIVE_PHOSPHORYLATION across HALLMARK and KEGG collections, together with GLYCOLYSIS and negative PURINE_METABOLISM enrichment, parallels the multi-pathway metabolic reprogramming causally attributed to AD keratinocytes by Mendelian randomization, suggesting cell-autonomous metabolic adaptation rather than bystander dysregulation. FOXC1 co-occurrence with negative E2F_TARGETS, MYC_TARGETS, and DNA_REPLICATION enrichment indicates proliferative quiescence, distinguishing Kcs9 from PCLAF-high proliferating KC subpopulations in AD and MKI67 + /TOP2A+ cycling states in psoriasiform dermatitis [30]. This suprabasal identity is independently anchored by CA2 localization to suprabasal differentiated keratinocytes in AD epidermis [27], with carbonic anhydrase family causal relevance reinforced by Mendelian randomization evidence identifying CA4 as an independent AD risk gene. Jointly, these signals support a unified model in which Kcs9 represents a suprabasal, barrier-repair–oriented keratinocyte state deploying paracrine growth factor signaling and metabolic reprogramming as primary adaptive responses to AD inflammatory stress—rather than hyperproliferation or direct cytokine amplification—thereby generating specific, experimentally testable predictions regarding its functional role.

In this context, elevated CA2 could represent (i) a potential functional contributor to maintaining intracellular/extracellular acid–base balance during stress, (ii) a proxy for a broader keratinocyte stress–remodeling state, or (iii) both, depending on the specific biology and lineage position of the Kcs9-like population. Our docking/MD analyses are consistent with CA2 being not only a computationally inferred state-associated gene but also a potentially tractable in silico target for baicalin; however, the direction and magnitude of functional modulation, and whether modulation would affect barrier-repair programs or influence inflammatory signaling contexts, require targeted experimental validation.

### Translational implications: A path from epidermal state to testable intervention

Our results suggest a translational path centered on epidermal accessibility, state-based stratification, and mechanistic complementarity to immune-targeted therapies—without implying immediate clinical efficacy. First, because the epidermis is directly accessible, an epidermal target such as CA2 and a keratinocyte state such as Kcs9 conceptually support exploration of topical or localized delivery strategies, which may reduce systemic exposure and enable direct modulation of barrier-adjacent programs. Second, CA2-high status or a Kcs9-like transcriptional signature could potentially serve as computationally derived hypothesis for a biomarker framework for patient stratification or pharmacodynamic readouts (e.g., identifying individuals whose disease is dominated by a keratinocyte stress–remodeling state), though this requires validation in independent cohorts and longitudinal sampling. Third, the keratinocyte-centered mechanism suggested by our computational findings may complement established immune-directed approaches by targeting the barrier–inflammation feedback loop from the epidermal side, which may be relevant to residual disease activity that can persist despite cytokine blockade in some patients.

The computational nominations presented here define a prioritized but unvalidated hypothesis requiring stepwise experimental corroboration before any mechanistic or therapeutic conclusions can be drawn. We propose a staged validation framework: first, CA2 expression and Kcs9-like signatures should be confirmed in independent AD cohorts and spatially localized via spatial transcriptomics or multiplex imaging. Second, direct baicalin–CA2 binding should be evaluated using orthogonal biophysical assays (SPR/MST/ITC) and cellular target engagement approaches (CETSA/DARTS), with CA2 enzymatic activity measured to establish mechanistic directionality. Third, barrier-relevant functional readouts should be assessed in Kcs9-enriched keratinocyte model systems. Finally, if in vitro engagement is confirmed, epidermal-compatible baicalin formulations should be evaluated in established AD animal models.

### Generalizability of the framework and relevance of the baicalin–CA2–Kcs9 axis beyond AD

The computational framework presented here—integrating pharmacogenomic target mining, dual machine-learning feature selection, single-cell state–resolved perturbation prioritization, and structure-based feasibility assessment—is not inherently disease- or drug-specific, and is in principle applicable to the systematic identification of other molecule–target–cell state axes in AD. The same workflow could in principle be applied to other bioactive compounds reported in AD (e.g., quercetin, berberine) against the same scRNA-seq reference atlas to nominate additional molecule–target–cell state axes, or extended to non-keratinocyte compartments such as fibroblasts or mast cells, with relatively modest methodological modification.

Regarding cross-disease relevance, the baicalin–CA2–Kcs9 axis may warrant investigation in other skin diseases characterized by epidermal barrier disruption and keratinocyte dysregulation. Psoriasis is a particularly relevant context: CA2 expression has been reported in psoriatic lesional skin, albeit at lower levels than in AD [27], suggesting that CA2-associated keratinocyte states may reflect a shared epidermal stress–remodeling response modulated by inflammatory context. Other conditions involving barrier dysfunction—including ichthyoses and allergic contact dermatitis—represent additional candidates addressable using publicly available scRNA-seq datasets. We acknowledge that cross-disease validation remains an important direction for future work.

## Limitations and future directions

Several limitations warrant acknowledgment. Most importantly, the present study is entirely computational: no direct baicalin treatment, CA2 inhibition assay, keratinocyte experiment, AD model, or target-engagement assay was performed. The proposed “baicalin–CA2–Kcs9” axis therefore represents a computationally derived, hypothesis-generating framework and should not be interpreted as a demonstrated therapeutic mechanism or confirmed biological interaction. All conclusions are inferred from database mining, transcriptomic association analyses, and in silico modeling, each of which precludes causal or mechanistic inference without experimental corroboration.

Beyond this fundamental scope limitation, additional technical constraints apply. Target nomination and ML prioritization depend on database coverage and analytical choices; convergence on CA2 increases confidence but cannot exclude false positives or targets absent from queried resources. The scRNA-seq datasets carry inherent technical noise (dropout effects, residual batch-correction artifacts) that may affect CA2 expression precision and Kcs9-like state reproducibility. Additionally, formal control for keratinocyte differentiation state and cell cycle variation was not performed; CA2 expression may partly reflect differentiation-associated gradients rather than disease-specific transcriptional changes. The identity of Kcs9 as a defined keratinocyte subtype remains incompletely resolved and requires marker-level validation against known epidermal states. Augur and scDist reflect expression-based stratification rather than pharmacological perturbation, and thus cannot establish whether baicalin modulates CA2 activity or the direction of any functional effect. Finally, docking and 100-ns MD with MM-PBSA/FEL support a plausible baicalin–CA2 complex in silico, but are subject to known constraints: MM-PBSA underestimates entropic contributions, force-field parameterization of baicalin introduces uncertainty, and 100-ns sampling may miss slow conformational transitions. Furthermore, the scRNA-seq dataset used in this study (GSE153760) lacks accompanying metadata on lesional versus non-lesional status, disease severity scoring, and treatment status at the time of sampling; these unmeasured variables may influence CA2 expression levels and keratinocyte state composition, and their absence limits the interpretability of CA2-associated signals with respect to disease stage or therapeutic context. Future studies incorporating severity-stratified or lesional/non-lesional-resolved scRNA-seq datasets would strengthen the biological interpretation of the baicalin–CA2–Kcs9 axis identified here.

A detailed, staged experimental validation roadmap—spanning transcriptomic confirmation, direct target engagement assays, keratinocyte functional readouts, and in vivo assessment—is outlined in the Discussion, and represents the logical next step for translating these computational nominations into testable biological hypotheses.

## Conclusion

**Fig 7:**
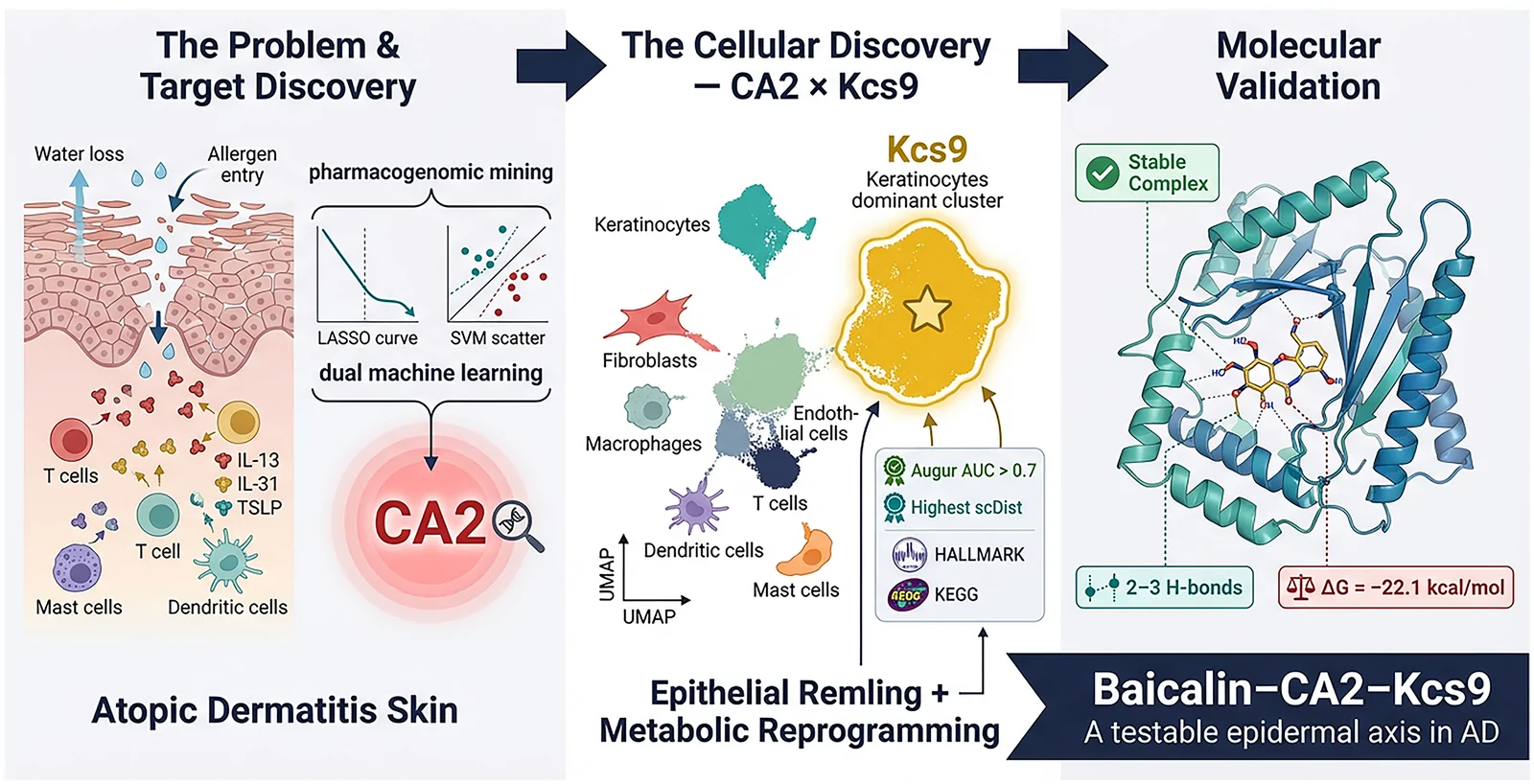
Graphical abstract summarizing the baicalin–CA2–Kcs9 framework in atopic dermatitis. Pharmacogenomic target mining combined with dual machine-learning feature selection (LASSO and SVM-RFE) identified CA2 as a convergent candidate target of baicalin in atopic dermatitis (AD). Single-cell RNA sequencing analysis localized CA2-associated transcriptional differences to keratinocytes, the cell type with the strongest CA2-stratified separability across major skin cell populations. Sub-clustering of keratinocytes into 14 states and dual perturbation prioritization using Augur (AUC > 0.7) and scDist nominated Kcs9 as the most CA2-associated transcriptionally divergent keratinocyte subpopulation, accompanied by enrichment signals in epithelial remodeling and metabolic programs. Molecular docking and 100-ns MD simulations supported a computationally plausible baicalin–CA2 complex (2–3 hydrogen bonds; ΔG = −22.1 kcal/mol). Together, these results nominate a testable “Baicalin–CA2–Kcs9” epidermal axis in AD.

In conclusion, by integrating pharmacogenomic target mining, dual machine-learning feature selection, single-cell state–resolved perturbation analyses, and structure-based molecular modeling, we identified a computationally coherent ‘baicalin–CA2–Kcs9’ candidate axis in atopic dermatitis (Fig 7). CA2 emerged as a convergent epidermal target localized to keratinocytes, with the Kcs9 subpopulation exhibiting the strongest CA2-stratified transcriptomic separability and transcriptomic distance, accompanied by co-enrichment signals spanning metabolic reprogramming, epithelial junction and barrier remodeling, and proliferative quiescence. Molecular docking and dynamics simulations supported a computationally plausible baicalin–CA2 interaction with an estimated favorable binding free energy in silico. This framework provides a concise, testable hypothesis linking a candidate therapeutic molecule to a specific disease-relevant keratinocyte state, offering a foundation for targeted experimental validation and epidermal-focused stratification strategies in AD.

## Supporting information

S1 Table Gene set enrichment analysis (GSEA) results for CA2-stratified and Kcs9-stratified comparisons across HALLMARK and KEGG collections. This table contains four sheets: (1) CA2_HALLMARK — HALLMARK GSEA results for CA2-high versus CA2-low cells across all major cell types, reporting gene set ID, description, set size, enrichment score, normalized enrichment score (NES), nominal p-value, adjusted p-value (BH-FDR), q-value, rank at maximum enrichment, leading-edge summary, and core enrichment genes; (2) CA2_KEGG — KEGG GSEA results for the same CA2-stratified comparison; (3) Kcs9_HALLMARK — HALLMARK GSEA results for CA2-high versus CA2-low cells within the Kcs9 subpopulation; (4) Kcs9_KEGG — KEGG GSEA results for CA2-high versus CA2-low cells within the Kcs9 subpopulation. All reported gene sets passed FDR < 0.05 unless otherwise noted.
(XLSX)
